# Antioxidant and hepatic protective effects of lotus root hot water extract with taurine supplementation in rats fed a high fat diet

**DOI:** 10.1186/1423-0127-17-S1-S39

**Published:** 2010-08-24

**Authors:** Huan Du, Xu Zhao, Jeong-Soon You, Ji-Yeon Park, Sung-Hoon Kim, Kyung-Ja Chang

**Affiliations:** 1 Department of Food and Nutrition, Inha University, Incheon, Korea; 2 Department of Chemistry, Konkuk University, Seoul, Korea

## Abstract

**Background:**

*Nelumbo nucifera*, known as sacred lotus, is a well-known medicinal plant and this lotus root is commonly used as food compared to different parts of this plant. This study was conducted to investigate the antioxidant and hepatic protective effects of lotus root hot water extract with taurine supplementation in high fat diet-induced obese rats.

**Methods:**

Thirty-two  male Sprague-Dawley rats (4-week-old)  were randomly divided into four groups (n=8) for 6 weeks (normal diet, N group; high fat diet, HF group; high fat diet + lotus root hot water extract, HFR group; high fat diet + lotus root hot water extract + taurine, HFRT group). Lotus root hot water extract was orally administrated (400mg/kg/day) to HFR and HFRT groups and the same amount of distilled water was orally administered to N and HF groups. Taurine was supplemented by dissolving in feed water (3% w/v).

**Results:**

The activities of glutamate oxaloacetate transaminase and glutamate pyruvate transaminase in serum were lower in HFR and HFRT groups compared to HF group. Thiobarbituric acid reactive substance contents in all groups fed a high fat diet were higher compared to N group.  The activities of hepatic antioxidant enzymes were higher in HFR and HFRT groups compared to HF group.

**Conclusions:**

These results suggest that lotus root hot water extract with taurine supplementation shows antioxidant and hepatic protective effects in high fat diet-induced obese rats.

## Background

High fat diet leads to an increase in oxidative stress levels [1] which is related to many human diseases such as cancer, ischemia, failures in immunity and endocrine functions [2]. It is also believed that oxidative stress is induced by reactive oxygen species (ROS) as its primary factor in aerobic organisms. ROS formed during normal metabolic processes can easily initiate the peroxidation of membrane lipids, leading to the accumulation of lipid peroxides [3,4]. In addition, high fat diets may induce accumulation in liver (non alcoholic fatty liver disease) [5].

Plants are widely used to delay the oxidation process as antioxidants and free radical scavengers. Lotus root was considered to possess a strong astringent herb in Tradition Chinese Medicine which helps to treat all manners of bleeding and haematemesis [6]. It has been reported  that lotus root has the activities of hypoglycemic, antifungal, antiinflammatory, antipyretic and antianxiety properties [7].  A previous study showed that various extracts of lotus rhizome exhibited higher antioxidant activity[8]. Taurine, a free amino acid, plays various physiological roles such as antiinflammation, neuroprotector, antidiabetes, immune regulation, etc. [9]. It has been reported that taurine possesses antioxidant properties [10,11], which is contributed by scavenging ROS [12] and reducing TBARS[13], and possesses the activity of hepatic protection [14].

Because lotus root has high antioxidant activity, it seems that lotus root possesses activity of hepatic protection. However, there are few papers about the effect of lotus root on hepatic protection. Therefore, this study was conducted to evaluate antioxidant and hepatic protective effects of lotus root hot water extract with taurine supplementation in rats fed a high fat diet.

## Methods

### Animals and diet

Three-week old male Sprague-Dawley rats were purchased from Hyundai-Bio (Anseong, Korea). All rats were kept at laboratory animal housing at Inha University following the recommendation of the Guide for the Care and Use of Laboratory Animals (Resources 1996) with constant 12 h light and dark cycle (AM 09:00 ~ PM 09:00), controlled temperature (23 ± 1℃) and humidity (55 ± 10%). Following one week of acclimatization with a pelletized commercial diet, rats were randomly divided into four groups (n = 8) for a period of 6 weeks (normal diet, N group; high fat diet, HF group; high fat diet + lotus root hot water extract, HFR group; high fat diet + lotus root hot water extract + taurine, HFRT group). Lotus root hot water extract was orally administrated (400mg/kg/day) to HFR and HFRT groups, and the same amount of distilled water was orally administered to N and HF groups. Taurine was supplemented by dissolving in feed water (3% w/v). Diet and water intakes were measured everyday and body weight was measured once every two days. The composition of the experimental diet was based on AIN76 [15] as shown in Table 1.

**Table 1:** Composition of experimental diets (g/100g diet)

Ingredients	Experimental diets	
	
	Normal Diet	High Fat Diet
Choline bitartrate	0.2	0.2
DL-Methione	0.3	0.3
Vitamin mixture	1	1
Mineral mixture	3.5	3.5
Cellulose	5	5
Sucrose	50	40
Corn starch	15	10
Casein	20	20
Bean oil	5	5
Lard	0	15

Carbohydrate (kcal / 100g)	260	200
Protein (kcal / 100g)	85	85
Fat (kcal /100g)	45	180
Total calories (kcal / 100g)	390	465

Vitamin mixture: AIN-76 Vitamin Mixture (g/kg);Thiamin hydrochloride 600mg, Riboflavin 600mg, pyridoxine hydrochloride 700mg, nicotinic acid 3g, D-calcium pantothenate 1.6g, Folic acid 200mg, D-biotin 20mg, Cyanocobalamin 1mg Retinyl palmitate pre-mix (250,000IU/g) 1.6g, DL-alpha-tocopherol acetate (250IU/g) 20g, cholecalciferol (400,000IU/g) 250mg, Menaquinone 5mg Sucrose, finely powdered 972.9g; Mineral mixture: AIN-76 Mineral Mixture (g/kg); Calcium phosphate dibasic 500g, Sodium chloride 74g, Potassium citrate monohydrate 220g, Potassium sulfate 52g, Magnesium oxide 24g, Manganous carbonate (43~48%Mn) 3.5g, Ferric citrate (16~17%Fe) 6g, Zinc carbonate (70% ZnO) 1.6g, Cupric carbonate (53~55% Cu) 0.3g, Potassium iodate 0.01g, Sodium selenite 0.01g, Chromium potassium sulfate 0.55g, Sucrose finely powdered 118g.

### Preparation of lotus root hot water extract

A dried block of lotus root was purchased from Seonwon Temple (Ganghwa-gun, Incheon, Korea). Previous studies recommend that functional components should be extracted at 80~90℃ [16,17]. In this study, lotus samples were extracted by water at 90℃ with a solid-liquid ratio of 2.5g/100ml for 2 hours. After vacuum filtration, the extract was introduced into a rotary evaporator (Büchi Rotavapor, Büchi Laboratoriums Teknik, Flawil, Switzerland). The concentrated liquid was dried by using a freeze dryer (Iilshin, Seoul, Korea). The brown powder was obtained with 30.3% extraction yield and then it was stored at -20℃ until application.

### Sampling and tissue preparation for biochemical analysis

The animals were fasted for 12 hours before sacrifice. Blood was collected from the heart, and serum was obtained by centrifugation at 3000rpm for 20 minutes. The epididymal fat (E-fat) and retroperitoneal fat(R-fat) were weighed. Some liver tissue was removed from the rats for histological photograph. The procedure for preparation of cytosol and mitochondria was partially modified from the method reported by Lee [18]. Approximately 5 grams of minced liver tissue was mixed with 10ml cold potassium phosphate buffer (154mM KCl, 50mM Tris-HCl, 1mM EDTA buffer, pH 7.4) in a Potter homogenizer (Sartorius, Goettingen, Germany) with gap from 0.095mm to 0.115mm. The homogenate was centrifuged for 10 min at 1000g to remove the precipitate, 1ml of above supernatant solution was stored for analyzing TBARS, and the remaining supernatant solution was centrifuged for 15min at 12,000g to remove the cell debris by using a centrifuge (Beckman J25-I, Beckman, USA). The precipitate was mitochondria, which was sonicated for 30s with a dismembrator (Fisher 300, Fisher Scientific, USA); the cytosol was obtained by centrifuging the supernatant solution for 60 min at 100,000g to remove the microsome by using an ultracentrifuge (T2080, Kontron Instruments, USA). All procedures were carried out at 0~4℃. After processing, all of the samples were immediately frozen in liquid nitrogen, and then stored at -70℃ until application (Operon, Korea).

### GOT and GPT activities

Activities of serum glutamic oxaloacetic transaminase (GOT) and glutamic pyruvic transaminase (GPT) were analyzed by using UV rate method [19]. After thawing for 30 minutes at room temperature, both were analyzed using an automatic analyzer (BPC BioSed srl, Rome, Italy). All of the results were expressed as IU/l serum.

### TBARS contents and SOD, GSH-Px and catalase activities

Serum and hepatic lipid peroxide contents were analyzed using the method of thiobarbituric acid (TBA) described by Buege and Aust [20]. The standard curve was prepared by 1,1,3,3-tetraethoxypropane from 0 to 50µmol/tube (R-square = 0.9999).

Cytosolic SOD activity was analyzed based on epinephrine autoxidation using Misra’s method [21]. The SOD activity was expressed as inhibiting the oxidation of epinephrine by 50% is equal to 1 unit.

GSH-Px activity was analyzed using the method described by Tappel [22]. Cumene hydroperoxide was used as the peroxide substrate in this method. The decrease in absorbance of NADPH was measured at 340nm by spectrometer (HP8453, Hewlett Packard, USA). One unit defined as 1 nmol of NADPH was converted to NADP^+^ per minute at 37℃ and pH 7.6. GSH-Px activity was calculated using NADPH molar extinction coefficient of 0.00622µM-1cm-1, and the results were expressed as IU per milligram protein.

Catalase activity was analyzed based on hydrogen peroxide to release oxygen and water under the catalytic influence of catalase [23]. One unit was defined as the amount of enzyme which decomposed 1 nmol of H_2_O_2_ per minute at 25 and pH 7.0. Catalase activity is calculated using H_2_O_2_ molar extinction coefficient of 43.6M^-1^cm^-1^ which expressed as IU per milligram protein.

### Hepatic morphology

Hepatic morphology was analyzed based on the paraffin method using a light microscope. Fresh tissues were fixed immediately in Bouin’s solution for 6-12 hours and then fixed tissue was washed under running water. After being dehydrated through different grades of alcohol, the tissues were embedded in paraffin block at 60°C. Eight µm sections were cut and mounted on glass slides coated with an egg albumin, and then the paraffin was removed with xylem and alcohol. The glass slides were stained with hematoxylin and eosin. After being dehydrated and cleared by alcohol and xylem, the glass slides were mounted in Canada Balsam. Photomicrographs were taken with a Zeiss Axiolab light microscope equipped with a Nikon Microflex HFX microscope camera.

### In vitro assay DPPH radical scavenging activity

l,l-Diphenyl-2-picryl-hydrazyl (DPPH) radical scavenging activity was analyzed using the method described by Nanjo, Goto *et al*. [24]. Sixty µl methanol solutions of sample with various concentrations were added to 60µl DPPH in methanol solution. After mixing vigorously for 10 sec, the solution was then transferred into a 100 μl Teflon capillary tube, and the scavenging activity of each enzymatic extract on DPPH radical was measured using an ESR spectrometer. A spin adduct was measured on a JES-FA ESR spectrometer (JEOL LTD., Tokyo, Japan) exactly 2 min later. Measurement conditions: central field, 3475 G; modulation frequency, 100 kHz; modulation amplitude, 2 G; microwave power, 5 mW; gain, 6.3×105 and temperature, 298 K.

### Alkyl radical scavenging activity

Alkyl radical scavenging activity was analyzed using the method described by Hiramoto *et al*. [25]. Alkyl radicals were generated by 2, 2'-azobis (2-amidinopropane) hydrochloride (AAPH). The phosphate buffered saline (pH 7.4) reaction mixtures containing 10 mM AAPH, 10 mM α-(4-pyridyl-1-oxide)-N-t-butylnitrone (4-POBN) and indicated concentrations of samples, which were incubated in a water bath for 30 minutes at 37°C and then transferred to a capillary tube. The spin adduct was recorded on a JES-FA ESR spectrometer (JEOL LTD., Tokyo, Japan). Measurement conditions: central field, 3,475 G; modulation frequency, 100 kHz; modulation amplitude, 2 G; microwave power, 1 mW; gain, 6.3 × 105; and temperature, 298 K.

### Hydroxyl radical scavenging activity

Hydroxyl radical scavenging activity was analyzed using the method described by Rosen and Rauckman [26]. Hydroxyl radicals were generated by iron-catalyzed Haber–Weiss reaction (Fenton driven Haber–Weiss reaction) and the generated hydroxyl radicals rapidly reacted with nitrone spin trap 5, 5-dimethyl-1-pyrroline-N-oxide (DMPO). The resultant DMPO-OH adduct was detectable with an ESR spectrometer. In brief, 0.2ml sample with various concentrations was mixed with 0.2ml DMPO (0.3M), 0.2ml FeSO4 (10mM) and 0.2 ml H2O2 (10mM) in a phosphate buffer solution (pH 7.2), and then introduced into 100 µl Teflon capillary tube. After 2.5 minutes, an ESR spectrum was recorded using a JES-FA ESR spectrometer (JEOL LTD., Tokyo, Japan). Measurement conditions: central field, 3475 G; modulation frequency, 100 kHz; modulation amplitude, 2 G; microwave power, 1 mW; gain, 6.3×105 and temperature, 298 K.

### Statistical analysis

Data were analyzed for significant difference by one-way analysis of variance followed by Duncan’s multiple range tests at a p<0.05. The 50% inhibition concentration (IC_50_) was calculated using probit regression analysis. All analyses were performed using SPSS 17.0 program.

## Results and discussion

### Body weight and adipose tissue weight

The final body weight of HF group was significantly higher compared to other groups (Table 2) and E-fat and R-fat weights of HF group were significantly higher compared to other groups. These results suggest that lotus root alone or with taurine supplementation has beneficial effect on lowering body weight and adipose tissue weight.

**Table 2:** Effect of lotus root hot water extract with taurine on body and adipose tissue weights

Group	Initial Weight			Final Weight			E-fat			R-fat		
N	153.6	±	5.2^NS^	352.7	±	7.8^a^	4.9	±	0.5^a^	4.5	±	0.9^a^
HF	153.0	±	3.3	411.0	±	15.3^b^	8.9	±	0.3^b^	12.0	±	1.5^b^
HFR	153.1	±	4.3	353.5	±	11.5^a^	6.2	±	0.7^a^	5.2	±	0.8^a^
HFRT	153.8	±	3.9	370.8	±	16.6^a^	6.1	±	0.9^a^	6.1	±	1.7^a^

E-fat: epididymal fat; R-fat: retroperitoneal fat; Values are mean ± SE; Values with different superscripts within the column are significantly different at p<0.05 by Duncan’s multiple range test.

### GOT and GPT activities

The activities of GOT and GPT in HFLT group were significantly lower than in HF group (Table 3). Since GOT and GPT exist primarily in hepatocytes and occur in trace amounts in serum, which are released into serum during hepatic damage. Thus, these results suggest that lotus root hot water extract with taurine supplementation may prevent hepatic steatosis.

**Table 3:** Effect of lotus root hot water extract with taurine on serum GOT and GPT activities

Group	GOT (IU/l)			GPT (IU/l)		
N	227.5	±	11.4^b^	84.3	±	7.5^a^
HF	292.4	±	8.5^c^	113.4	±	4.4^b^
HFR	260.6	±	17.7^bc^	101.7	±	4.7^b^
HFRT	176.1	±	7.6^a^	84.1	±	4.8^a^

GOT: glutamate oxaloacetate transaminase; GPT: glutamate pyruvate transaminase; Values are mean ± SE; Values with different superscripts within the column are significantly different at p<0.05 by Duncan’s multiple range test.

### TBARS contents and SOD, GSH-Px and catalase activities

The results of TBARS contents and SOD, GSH-Px and catalase activities were shown in Table 4. The serum and hepatic TBARS contents in the HF, HFR and HFRT groups were higher than in the N group in both serum and liver, which indicates that oxidative stress is increased in rats fed a high fat diet.

**Table 4:** Effect of lotus root hot water extract with taurine on TBARS  and antioxidant enzymes

Group	Hepatic TBARS (nmol/mg protein)			Serum TBARS (µmol/L serum)			SOD (IU/mg protein)			GSH-Px (IU/mg protein)			Catalase (IU/mg protein)		
N	26.4	±	2.5^a^	2.97	±	0.71^a^	9.96	±	0.23^c^	0.10	±	0.02^a^	150.83	±	17.61^b^
HF	55.4	±	4.2^b^	4.92	±	0.41^b^	2.26	±	0.53^a^	0.10	±	0.02^a^	82.94	±	11.22^a^
HFR	43.5	±	6.9^ab^	5.16	±	0.68^b^	6.54	±	1.18^b^	0.43	±	0.13^b^	154.24	±	16.72^b^
HFRT	51.3	±	15.0^ab^	4.91	±	0.65^b^	3.06	±	0.36^a^	0.30	±	0.03^ab^	159.49	±	12.72^b^

TBARS: thiobarbituric acid reactive substances; SOD: superoxide dismutase; GSH-Px: glutathione peroxidise; Values are mean ± SE; Values with different superscripts within the column are significantly different at p<0.05 by Duncan’s multiple range test

SOD and GSH-Px activities were significantly higher in HFR group compared to HF group. Catalase activity was significantly higher in HFR and HFRT groups compared to HF group.

### Hepatic morphology

**Figure 1:**
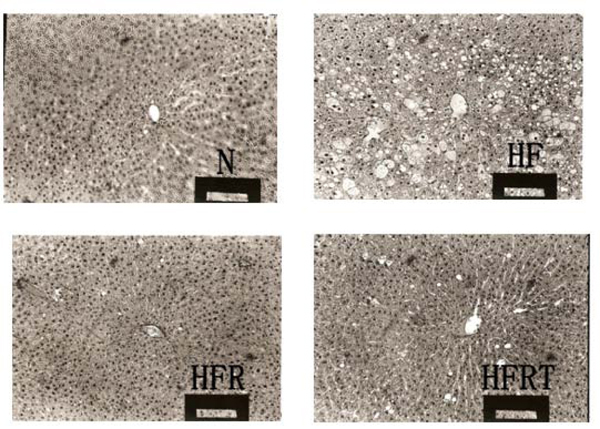
**Light micrographs of hepatocytes** in rats fed a normal rats’ diet or a high fat rats’ diet with supplementation of lotus root hot water extract or lotus root hot water extract and taurine. Magnification × 100. The photograph exhibited that formation of lipid droplets was suppressed in the HFR and HFRT groups, whereas rats in the HF group showed noticeable fatty liver. N, normal diet group; HF, high fat diet group; HFR, high fat diet + lotus root hot water extract group; HFRT, high fat diet + lotus root hot water extract + taurine group.

Hepatic histological photograph was shown in Figure 1 with distinguishable hepatic cells, central vein, and portal triad. The photograph exhibited that HFR and HFRT groups suppressed formation of lipid droplets, whereas the HF group showed noticeable fatty liver. From these results, it seemed that lotus root hot water extract alone or with taurine supplementation inhibits fat accumulation in liver.

### Free radical scavenging activity

The radical scavenging activities of lotus root hot water extract and taurine *in vitro* were focused on DPPH, alkyl and hydroxyl radical scavenging properties by using an electron spin resonance (ESR). The results were shown in Table 5. DPPH radical is a stable radical which gives the results shown that the lotus root hot water extract on DPPH radical scavenging activity was highest among the groups when the IC_50_ was 0.18mg/ml. The effects of lotus root hot water extract of hydroxyl and alkyl radical scavenging activities were highest among groups with an IC_50_ of 2.01mg/ml and 0.12mg/ml, respectively. The decrease of ESR signals of DPPH, alkyl and hydroxyl radical activities showed the dose-dependent activity of lotus root hot water extract (data not shown). Vitamin C was also measured as positive controls with their IC_50_ were 0.003mg/ml, 0.011mg/ml and 0.021mg/ml for DPPH, alkyl and hydroxyl radical scavenging activity, respectively. These results suggest that lotus root hot water extract possesses high free radical scavenging activity *in vitro*.

**Table 5:** IC_50_ of DPPH, hydroxyl, alkyl radical scavenging in lotus root hot water extract and taurine

IC50 (mg/ml)	DPPH	Alkyl	Hydroxyl
R	0.18	0.12	2.01
T	X	X	X
R1:T1	0.45	1.99	3.25
R1:T6	3.39	X	X
R1:T12	X	X	X
Vit C	0.03	0.01	0.02

X: result larger than 4mg/ml; R: lotus hot water extract; T: taurine; Vit C: vitamin C; R1:T1:mixture ratio between lotus root hot water extract and taurine was 1:1; R1:T6: mixture ratio between lotus root hot water extract and taurine was 1:6; R1:T12: mixture ratio between lotus root hot water extract and taurine was 1:12

## Conclusions

In conclusion, lotus root hot water extract alone or combined with taurine supplementation has antioxidant activity both *in vivo* and *in vitro*. Also lotus root hot water extract with taurine supplementation shows hepatic protective effects in high fat diet-induced obese rats

## Abbreviations

℃: celsius degree; E-fat: epididymal fat; R-fat: retroperitoneal fat; GOT: glutamate oxaloacetate transaminase; GPT: glutamate pyruvate transaminase; TBARS: thiobarbituric acid reactive substances; SOD: superoxide dismutase; GSH-Px: glutathione peroxidise; X: result larger than 4mg/ml; R: lotus hot water extract; T: taurine; ESR: electron spin resonance

## Competing interests

The authors declare that they have no competing interests.

## Authors' contributions

HD carried out design, execution, data analysis, and manuscript preparation of the study. XZ, JSY, and JYP participated in design, sample assay, and analysis. The method of extracting lotus root was provided by SHK. KJC guided in all the study. All authors read and approved the final manuscript.
